# Endoplasmic reticulum stress disrupts placental morphogenesis: implications for human intrauterine growth restriction

**DOI:** 10.1002/path.4068

**Published:** 2012-09-28

**Authors:** Hong wa Yung, Myriam Hemberger, Erica D Watson, Claire E Senner, Carolyn P Jones, Randal J Kaufman, D Stephen Charnock-Jones, Graham J Burton

**Affiliations:** 1 Centre for Trophoblast Research, University of CambridgeCambridge, CB2 3EG, UK; 2 Epigenetics Programme, The Babraham Institute, Babraham Research CampusCambridge, CB22 3AT, UK; 3 Maternal and Fetal Health Research Group, School of Biomedical Sciences, University of ManchesterManchester, M13 9WL, UK; 4 Del E Webb Neuroscience, Aging and Stem Cell Research Center, Sanford/Burnham Medical Research Institute10901 North Torrey Pines Road, La Jolla, CA 92037-1062, USA; 5 Department of Obstetrics and Gynaecology, University of CambridgeCambridge, CB2 0SW, UK; 6 National Institute for Health Research, Cambridge Comprehensive Biomedical Research CentreCambridge, UK

**Keywords:** endoplasmic reticulum, stress, placental morphogenesis, intrauterine growth restriction, Igf2, Pdk1, Akt, mTOR

## Abstract

We recently reported the first evidence of placental endoplasmic reticulum (ER) stress in the pathophysiology of human intrauterine growth restriction. Here, we used a mouse model to investigate potential underlying mechanisms. *Eif2s1*^*tm1RjK*^ mice, in which Ser51 of eukaryotic initiation factor 2 subunit alpha (eIF2α) is mutated, display a 30% increase in basal translation. In *Eif2s1*^*tm1RjK*^ placentas, we observed increased ER stress and anomalous accumulation of glycoproteins in the endocrine junctional zone (Jz), but not in the labyrinthine zone where physiological exchange occurs. Placental and fetal weights were reduced by 15% (97 mg to 82 mg, *p* < 0.001) and 20% (1009 mg to 798 mg, *p* < 0.001), respectively. To investigate whether ER stress affects bioactivity of secreted proteins, mouse embryonic fibroblasts (MEFs) were derived from *Eif2s1*^*tm1RjK*^ mutants. These MEFs exhibited ER stress, grew 50% slower, and showed reduced Akt–mTOR signalling compared to wild-type cells. Conditioned medium (CM) derived from *Eif2s1*^*tm1RjK*^ MEFs failed to maintain trophoblast stem cells in a progenitor state, but the effect could be rescued by exogenous application of FGF4 and heparin. In addition, ER stress promoted accumulation of pro-Igf2 with altered glycosylation in the CM without affecting cellular levels, indicating that the protein failed to be processed after release. Igf2 is the major growth factor for placental development; indeed, activity in the Pdk1–Akt–mTOR pathways was decreased in *Eif2s1*^*tm1RjK*^ placentas, indicating loss of Igf2 signalling. Furthermore, we observed premature differentiation of trophoblast progenitors at E9.5 in mutant placentas, consistent with the *in vitro* results and with the disproportionate development of the labyrinth and Jz seen in placentas at E18.5. Similar disproportion has been reported in the *Igf2*-null mouse. These results demonstrate that ER stress adversely affects placental development, and that modulation of post-translational processing, and hence bioactivity, of secreted growth factors contributes to this effect. Placental dysmorphogenesis potentially affects fetal growth through reduced exchange capacity. Copyright © 2012 Pathological Society of Great Britain and Ireland. Published by John Wiley & Sons, Ltd.

## Introduction

Intrauterine growth restriction (IUGR) refers to a fetus that has failed to achieve its genetically determined potential size, and affects approximately 5–8% of human pregnancies worldwide. It is a major cause of neonatal morbidity and mortality, and also increases the risk of developing cardiovascular and metabolic diseases in adulthood. Predisposing factors include maternal smoking, under-nutrition, infection, and congenital malformations, but in the majority of cases utero-placental insufficiency is thought to be the cause. The placenta serves as the interface between a mother and her fetus, and is critical for fetal nutrition. Reduced placental growth is observed to precede fetal IUGR during human pregnancy (1, 2). Similarly, deletion of the placental-specific *Igf2* promoter in the mouse, *P0*, results in a smaller placenta that in turn restricts fetal growth in late gestation (3). Consequently, it is widely accepted that placental compromise is one of the major risk factors for the development of IUGR and has been described as ‘placental IUGR’ (4).

We recently identified endoplasmic reticulum (ER) stress as a feature of the placental pathophysiology in cases of human IUGR (5). The ER serves multiple functions, including the synthesis, post-translational modification, and trafficking of membrane and secreted proteins. Perturbation of ER homeostasis may result in misfolding or abnormal glycosylation of these proteins, which has the potential to reduce their biological activity. The accumulation of unfolded or misfolded proteins within the ER cisternae provokes ER stress and activation of the unfolded protein response (UPR). The UPR attempts to restore ER function by attenuation of protein translation, increasing the folding capacity and facilitating degradation of misfolded proteins (6). Protein synthesis can be regulated through phosphorylation of eIF2α at Ser51 by PKR-like endoplasmic reticulum kinase (PERK) (7), which then acts as a competitive inhibitor of eIF2B, thereby inhibiting translation initiation (8). If the mechanisms fail to reduce the stress, apoptosis ensues to eliminate the damaged cell.

Organs with major endocrine and/or exocrine activity are more vulnerable to ER stress due to their constitutively high level of protein translation. Indeed, both the pancreas and the placenta suffer low-grade ER stress under normal physiological conditions (9, 10). In both *Perk* knockout and *Eif2s1*^*tm1RjK*^ mutant mice (in which eIF2α cannot be inactivated by phosphorylation at Ser51 and hence protein synthesis is constitutively elevated), the β-pancreatic cells exhibit severe ER stress and increased apoptosis (11–13). ER stress is now widely accepted as a major aetiological factor for human type II diabetes (14).

In this study, we test whether ER stress is a sufficient cause of placental IUGR and further elucidate the underlying mechanisms using the *Eif2s1*^*tm1RjK*^ mutant mouse as an animal model.

## Materials and methods

### *Eif2s1*^*tm1RjK*^ mutant mice

*Eif2s1*^*tm1RjK*^ mutant C57BL/6 mice were generated as described by Scheuner *et al* (12). A total of 26 litters of mice containing 57 wild-type, 32 mutant, and 111 heterozygous animals was used. The average litter size was ∼8. All experiments were carried out in accordance with the UK Home Office Animals (Scientific Procedures) Act 1986. Placentas and fetuses were collected at E18.5 and immediately immersed in ice-cold phosphate buffered saline. After weighing, placentas were dissected into junctional zone-rich (Jz) and labyrinthine zone-rich (Lz) pieces, which were snap-frozen in liquid nitrogen. Tail tips were collected for genotyping.

### Mouse embryonic fibroblasts (MEFs)

MEFs were derived from E13.5 embryos as described in the Supplementary materials and methods.

### Trophoblast stem cells

A bioassay was used to follow trophoblast stem cell differentiation as described in the Supplementary materials and methods.

### EM

Ultrastructural analysis was performed as described in the Supplementary materials and methods.

### Western blot

Western blotting was performed as previously described (15), and the antibody details are described in the Supplementary materials and methods.

### Lectin staining

Staining was performed as described by Jones *et al* (16).

### RT-PCR analysis of *Xbp-1* mRNA spliced variants

The RT-PCR procedure was performed as previously described (15) and in the Supplementary materials and methods.

### TaqMan quantitative real-time RT-PCR

This was conducted as previously described (15).

### Stereology

The analysis was conducted as previously described in detail by Coan *et al* (17) and in the Supplementary materials and methods.

### Statistical analysis

Differences were tested using either the two-tailed Student's *t*-test or, when appropriate, the non-parametric Mann–Whitney *U*-test, with *p* < 0.05 being considered significant.

## Results

### ER stress is increased in the endocrine junctional zone, but not in the labyrinth, in homozygous *Eif2s1*^*tm1RjK*^ placentas, and is associated with reductions in both placental and fetal weight

The murine placenta is composed of two major zones with distinctive functions: the junctional zone (Jz), which has endocrine functions, and the labyrinthine zone (Lz), which performs nutrient and gas exchange. Ultrastructural analysis revealed that cells in the Jz contain more ER cisternae than those in the Lz, consistent with their high endocrine activity (Figure 1A and Supplementary Figure 1). We observed severe dilatation of cisternae in the Jz of *Eif2s1*^*tm1RjK*^ (A/A) placentas, indicating loss of homeostasis within the ER lumen (Figure 1A). To confirm that the dilatation resulted from ER stress, the Jz and Lz were separated by dissection and analysed for ER stress markers (Supplementary Figure 2A). There was an approximately two-fold increase in phosphorylation of Perk in the Jz of *Eif2s1*^*tm1RjK*^ placentas, but not in the Lz (Figure 1B). However, we observed no change in other ER stress markers, such as the ER chaperone proteins, Grp78 and 94, and splicing of *X-box binding protein-1* (*Xbp-1*) mRNA, in either zone, nor any increase in apoptosis (Supplementary Figures 2B–2D). These results revealed the existence of low-grade ER stress in the endocrine-active Jz of the *Eif2s1*^*tm1RjK*^ placenta.

**Figure:**
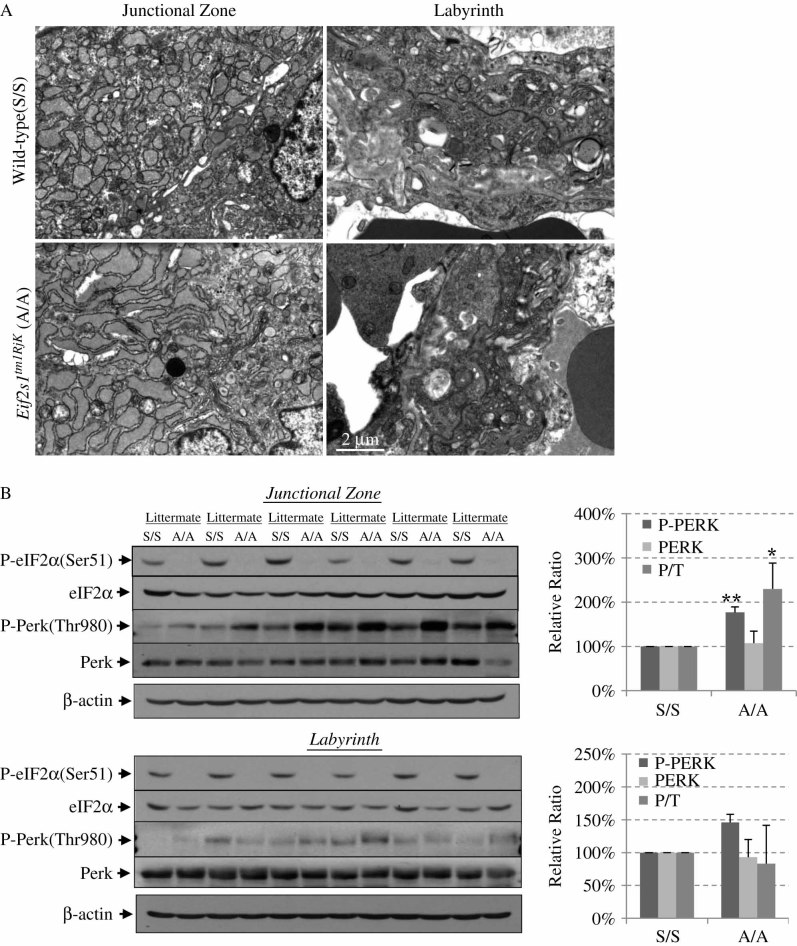
An increase of ER stress in the junctional zone (Jz) of the *Eif2s1*^*tm1RjK*^ (A/A) placenta. (A) Electron micrographs show severe dilation of ER cisternae in the spongiotrophoblast cells of the Jz in the *Eif2s1*^*tm1RjK*^ placenta compared with only moderate dilation in the wild-type. There are fewer ER cisternae in the syncytiotrophoblast of the Lz, and no dilation was observed in either genotype. (B) Placentas were dissected into Jz and Lz, and enriched tissue lysates for each zone were studied separately by western blotting analysis using specific antibodies against P-Perk (Thr980), total Perk, P-eIF2α and total eIF2α. β-Actin was used as a loading control. Experiments were carried out on a paired basis. (C) Densitometry of band intensity is expressed relative to wild-type (100%). Phosphorylation status is presented as the ratio between phosphorylated and total (P/T) protein. Data are presented as mean ± SEM; *n* = 6. *p≤0.05; **p≤0.01

**Figure:**
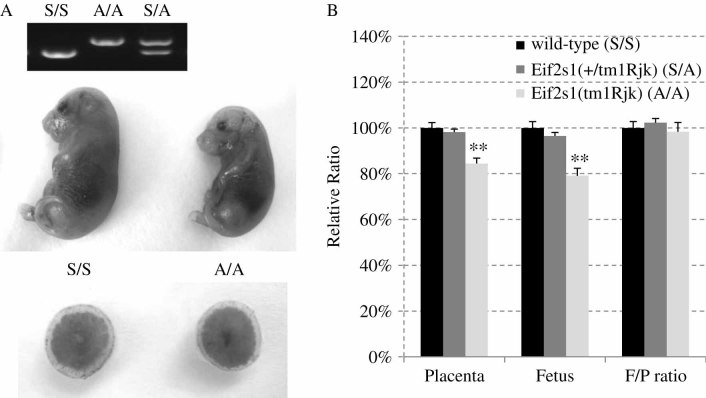
The *Eif2s1*^*tm1RjK*^ mice are associated with reduced placental and fetal growth. (A) Upper panel: the progeny of the mice as shown by PCR. Lower panel: *Eif2s1*^*tm1RjK*^ (A/A) homozygous embryo and placenta at E18.5 versus wild-type littermate. (B) Statistical analysis of fetal and placental weights from 195 pups from 29 litters. All data are expressed as percentage relative to wild-type (S/S, 100%). Bars indicate mean ± SEM. *p≤0.05

Despite having an ∼30% higher rate of protein synthesis (12), the *Eif2s1*^*tm1RjK*^ mutants exhibited an ∼15% reduction in placental weight (97 ± 17 mg to 82 ± 12 mg, *p* < 0.001) and ∼20% reduction in fetal weight (1009 ± 203 mg to 798 ± 182 mg, *p* < 0.001) at E18.5 (Figure 2). However, placental efficiency, which is calculated as the ratio between fetal and placental weight, remained unchanged (Figure 2B).

These results are consistent with our data from human IUGR placentas, in which ER stress was only observed in the syncytiotrophoblast, the major site for hormone synthesis, and not in the underlying progenitor cytotrophoblast layer (5).

### Anomalous accumulations of glycoproteins are found in the junctional zone of the *Eif2s1*^*tm1RjK*^ placenta

With few exceptions, the secreted proteins of eukaryotes are glycosylated within the ER before release. Specific glycan structures determine the *in vivo* properties of the proteins, including their circulatory lifetime, protease resistance, stability, and interaction with receptors. Perturbation of ER homeostasis disrupts the glycosylation process, and so has a potentially devastating impact on the functions of secreted proteins. For example, in influenza haemagglutinin, the presence of tunicamycin causes the precursor haemagglutinin to be misfolded and retained in the ER (18). A similar phenomenon is observed spontaneously in the *Eif2s1*^*tm1RjK*^ pancreas, where there is an anomalous accumulation of pro-insulin within β-cells (13). Therefore, we investigated whether there is an equivalent anomalous retention of secreted proteins in the Jz of *Eif2s1*^*tm1RjK*^ placentas. Placental sections were stained with four different lectins, [ConA (concanavalin A), e-PHA (*Phaseolus vulgaris*, erythrohaemagglutinin), DSA (*Datura stramonium* agglutinin), and STA (*Solanum tuberosum* agglutinin)]. Staining with each of the lectins was rare in the Jz of the wild-type (S/S) placentas. In contrast, strong staining was frequently observed in the cytoplasm of spongiotrophoblast cells of the Jz in the *Eif2s1*^*tm1RjK*^ (A/A) placentas (Figure 3 and Supplementary Figure 3), indicating anomalous intracellular accumulation of glycoproteins.

**Figure:**
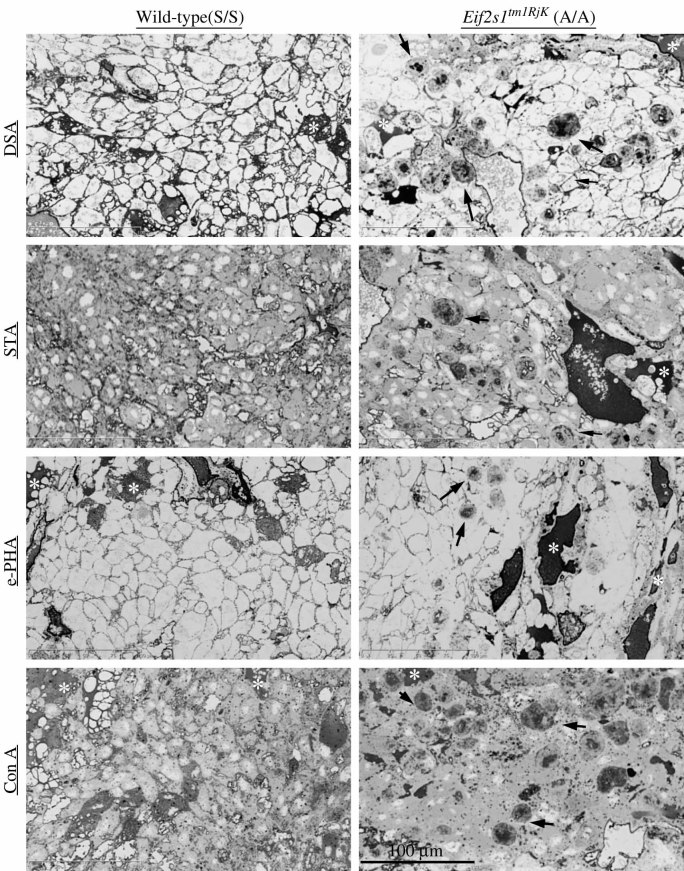
Anomalous accumulation of glycoproteins in the endocrine spongiotrophoblast cells of the Jz in the *Eif2s1*^*tm1RjK*^ placenta. Biotinylated lectins, ConA (concanavalin A), e-PHA (*Phaseolus vulgaris*, erythrohaemagglutinin), DSA (*Datura stramonium* agglutinin), and STA (*Solanum tuberosum* agglutinin) were used to stain the placental sections. Note that intensely dark staining accumulations in the rounded spongiotrophoblast cells (arrowed) are more common in the mutants. The cells must be distinguished from the irregularly shaped blood channels (asterisks), which also stain positively. All images taken at 20× magnification. ^*tm1RjK*^m

### Change in glycosylation profile in secreted proteins released from MEFs derived from *Eif2s1*^*tm1RjK*^ embryos

To further elucidate the impact of ER stress on glycosylation of secreted proteins, mouse embryonic fibroblasts (MEFs) were derived from E13.5 *Eif2s1*^*tm1RjK*^ and wild-type embryos. Characterization of *Eif2s1*^*tm1RjK*^ MEFs showed an increase of low-grade ER stress, with elevation of phosphorylated and total Perk, constant Grp78, and no alternative splicing of *Xbp-1* mRNA (Figure 4A), equivalent to the pattern seen in the Jz. The *Eif2s1*^*tm1RjK*^ MEFs grew ∼50% slower, with reduced phosphorylation of Akt at both Thr308 and Ser473, and of eIF4E binding protein 1 (4E-BP1) at Ser65, compared with wild-type MEFs, despite their intrinsically higher translation rate (Figures 4B and 4C).

**Figure:**
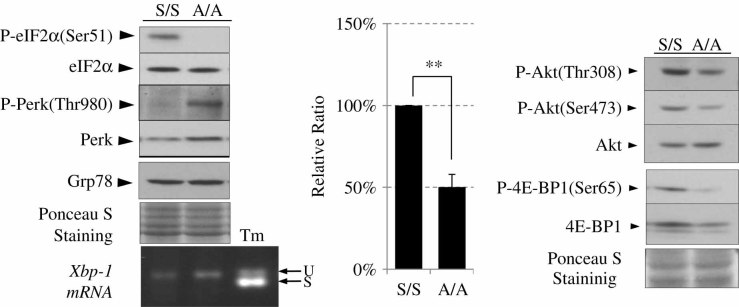
*Eif2s1*^*tm1RjK*^ MEFs exhibit higher ER stress, slower cell proliferation rate, and lower Akt–mTOR signalling than wild-type controls. (A, C) Both wild-type and *Eif2s1*^*tm1RjK*^ (A/A) MEFs were cultured in serum-free medium for 24 h. ER stress markers were investigated using antibodies against P-Perk, Perk, and Grp78, while Akt–mTOR signalling was examined using specific antibodies against P-Akt(Ser473), P-Akt(Thr308), Akt, P-4E-BP1 (Ser65), and 4E-BP1. Ponceau S staining was used as the loading control. For *Xbp-1* mRNA splicing, total RNA was isolated and RT-PCR was used to detect the spliced variants. Tunicamycin (Tm; 400 ng/ml) treatment for 24 h was used as a positive control. (B) MEFs were seeded at the same density and cultured for 4 days. The number of cells was counted using a haemocytometer. Data are presented as mean ± SEM, *n* = 4, relative to wild-type (S/S), which is presented as 100%. **pp≤0.01

To examine any change in glycosylation of the secreted proteins released from *Eif2s1*^*tm1RjK*^ MEFs, a concanavalin A column was used to isolate glycoproteins from the conditioned media before resolving by SDS-PAGE. Multiple bands were found to differ between wild-type (S/S) and *Eif2s1*^*tm1RjK*^ (A/A) (Figure 5A, arrowed). Similar changes were observed in positive controls, in which wild-type cells were treated with thapsigargin (Tg) or tunicamycin (Tm), suggesting that these changes likely result from ER stress.

**Figure:**
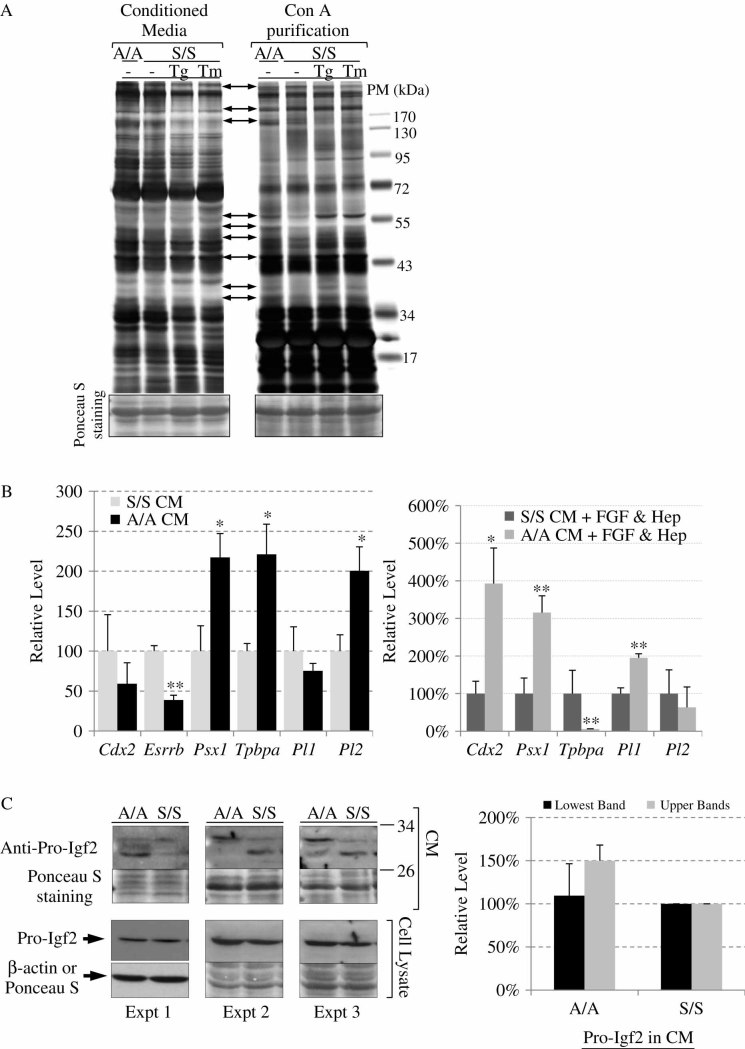
Increased ER stress in *Eif2s1*^*tm1RjK*^ (A/A) MEFs modulates the bioactivity of secreted proteins. (A) Alteration of protein glycosylation profile (arrows). Both *Eif2s1*^*tm1RjK*^ and wild-type MEFs, with or without 5 nm thapsigargin (Tg) or 10 ng/ml tunicamycin (Tm), were cultured in serum-free medium for 48 h in the presence of EGF. Conditioned media (CM) were concentrated and glycoproteins isolated using concanavalin A (ConA) lectin columns, followed by resolving by SDS-PAGE and silver staining. (B) Conditioned medium from *Eif2s1*^*tm1RjK*^ MEFs failed to maintain trophoblast stem cells in a multipotent state but this could be reversed by exogenous supplementation with FGF4 and heparin. Conditioned media prepared from wild-type and *Eif2s1*^*tm1RjK*^ MEFs were used to culture TS cells in the absence or presence of FGF4 and heparin. Total TS cell RNA was isolated, and qRT-PCR was used to assess the relative levels of different nuclear regulators of differentiation including the stem cell markers *Cdx2* and *Esrrb*, the early differentiation marker *Psx1*, the ectoplacental cone/spongiotrophoblast marker *Tpbpa*, and the giant cell markers *Pl1* and *Pl2. Sdha* and *Gapdh* were used as housekeeping genes. Data are presented as mean ± SEM from three independent experiments. (C) Altered processing of Igf2 in the *Eif2s1*^*tm1RjK*^ MEFs. Immunoblotting of conditioned media or cell lysate prepared from both *Eif2s1*^*tm1RjK*^ and wild-type MEFs. Antibodies specific for Pro-Igf2 were used to detect the proteins in both CM and cell lysates. β-Actin and Ponceau S staining were used to show equal loading. Left panel: three blots of pro-Igf2 in CM and cell lysates are presented to indicate the variation between experiments. The intensities of the lowest band and of the upper few bands were quantified separately to show the mobility shifting of pro-Igf2 due to changes in glycosylation. Right panel: data are presented as mean ± SEM from four independent experiments. *p < 0.05

A change in glycosylation pattern may affect the function of secreted proteins. Therefore, to investigate the bioactivity of secreted proteins released by *Eif2s1*^*tm1RjK*^ MEFs, a bioassay was established using trophoblast stem (TS) cells. TS cells require trophic factors in MEF conditioned medium for continued proliferation and maintenance of their progenitor state, and withdrawal promotes TS cell differentiation into trophoblast subtypes (19, 20). Therefore, conditioned media (CM) from MEFs were used to culture TS cells for 3 days. Compared with incubation with wild-type CM, TS cells cultured in *Eif2s1*^*tm1RjK*^ CM showed significantly increased levels of transcripts associated with trophoblast differentiation, such as *Psx1*, an early differentiation marker; *Tpbpa*, an ectoplacental cone/spongiotrophoblast marker; and *Pl2* (also known as *Prl3b1*), a giant cell marker. Correspondingly, there was a significant decrease in the stem cell marker, *Esrrb*, and a trend to a reduction in *Cdx2* (Figure 5B, left graph). However, supplementation with exogenous FGF4 and heparin in *Eif2s1*^*tm1RjK*^ CM prevented the trophoblast differentiation, as shown by increased *Cdx2* and reduced *Tpbpa* mRNA. This finding indicates that loss of signalling from trophic factors, such as FGF4 and TGFβ is the major cause of differentiation in TS cells cultured in *Eif2s1*^*tm1RjK*^ CM (Figure 5B, right graph).

Igf2 is a major stimulant of placental growth (21) and is released as a heavily glycosylated pro-form. Generation of active Igf2 requires extracellular enzymatic cleavage of the pro-protein. The degree of glycosylation determines its biological activity (22). The glycosylation pattern of pro-Igf2 was changed in the CM from *Eif2s1*^*tm1RjK*^ (A/A) MEFs compared with wild-type MEFs (Figure 5C). Immunoblotting of the cell lysate did not reveal any difference in cellular pro-Igf2 levels between the two cell types.

These data indicate that ER stress alters glycosylation of the secreted proteins and can modulate their bioactivity.

### Reduction of Pdk1–Akt–mTOR signalling in the *Eif2s1*^*tm1RjK*^ placenta

*Igf2*-deficient mice have smaller placentas and fetuses, and reduced Akt signalling, compared with wild-type littermates (Supplementary Figure 4) (23). This suggests that the loss of Akt signalling that we observed in *Eif2s1*^*tm1RjK*^ placentas may reflect loss of bioactivity of abnormally glycosylated pro-Igf2. Therefore, we analysed Akt–mTOR signalling in both the Jz and the Lz. There was a significant reduction of P-Pdk1 (Ser241), P-Akt (Ser473), P-Akt (Thr308), and P-4E-BP1 (Thr70) in both the Jz and the Lz of the *Eif2s1*^*tm1RjK*^ placentas (Figures 6A and 6B). Interestingly, we observed an approximately 50% reduction in total Akt in the Jz, but not in the Lz, of the *Eif2s1*^*tm1RjK*^ placenta. To determine whether the decrease in Akt was due to a reduction in *Akt* mRNA, we quantified by qRT-PCR all three Akt-encoding transcripts (*Akt1, Akt2*, and *Akt3*). As shown in Figure 6C, no change was observed in any of the three *Akt* transcripts, consistent with our previous reports that ER stress attenuates AKT protein translation (5, 15).

**Figure:**
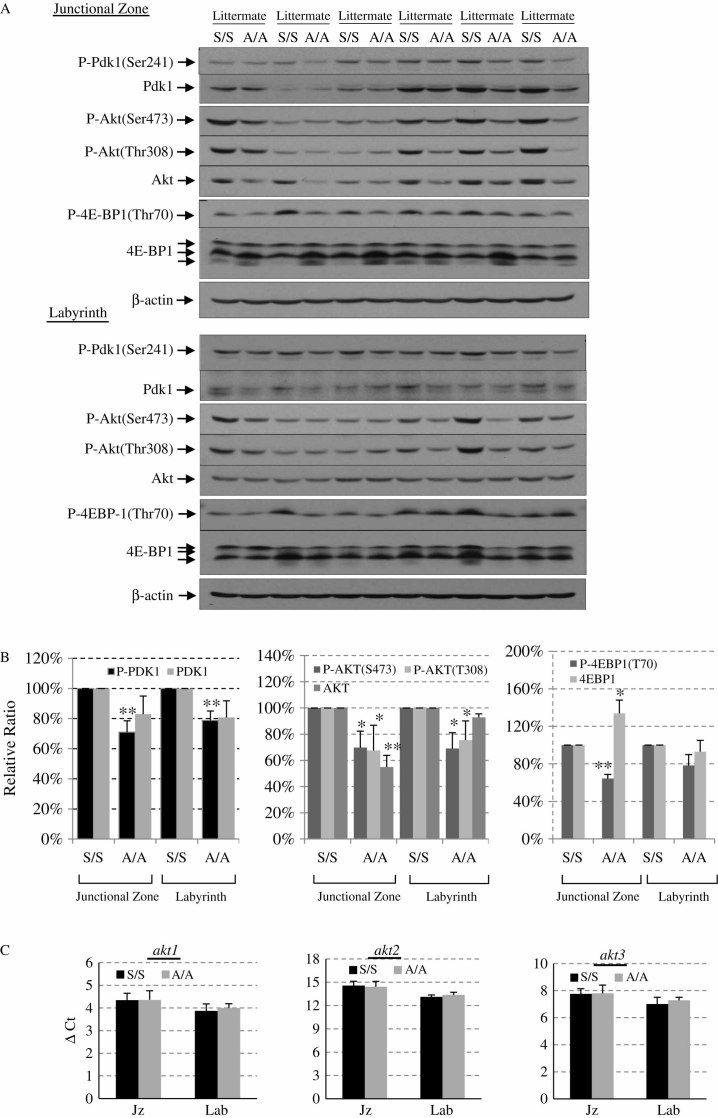
Down-regulation of Pdk1–Akt–mTOR signalling in the *Eif2s1*^*tm1RjK*^ placenta. (A) Western blotting was used to measure the phosphorylation and total level of P-Pdk1 (Ser241), Pdk1, P-Akt(Ser473), P-Akt(Thr308), Akt, P-4E-BP1 (Thr70), and 4E-BP1 using specific antibodies in both the Jz and Lz. (B) Densitometry of band intensity is expressed relative to wild-type (100%). Phosphorylation status is presented as the ratio between phosphorylated and total protein. *p≤0.05; **p≤0.01. (C) Real-time quantitative RT-PCR was used to measure the expression level of three *Akt* transcripts. Data are presented as mean ± SEM; *n* = 6. ΔCt was calculated from the cycle difference between target genes and the housekeeping gene, 18S

### Disruption of trophoblast differentiation by ER stress alters placental morphogenesis

Differentiation of trophoblast progenitor cells into specialized subtypes is crucial for the formation of the distinct functional zones of the placenta. Trophoblast lineage formation and stem cell maintenance are determined by cell-autonomous nuclear regulators, including *Cdx2* and *Esrrb*, and paracrine signals such as Fgf4 and Activin/Tgfβ/Nodal (19, 20, 24, 25). Loss of bioactivity in *Eif2s1*^*tm1RjK*^ MEF CM indicated a potential failure in the paracrine signals, thereby altering trophoblast differentiation and affecting placental morphogenesis. Therefore, we investigated placental structure using stereology to estimate the absolute volumes of the different zones in the *Eif2s1*^*tm1RjK*^ placentas at E18.5. As expected, the smaller *Eif2s1*^*tm1RjK*^ placentas showed an approximately 30% reduction in total volume (77 mm^3^ ± 3.9 mm^3^ versus 112.7 mm^3^ ± 2.8 mm^3^, *p* < 0.001, Figure 7A). The composition of the placenta was also significantly different, with a reduction in both the absolute volume of, and the proportion occupied by, the Lz (24.77 mm^3^ ± 1.59 mm^3^ versus 46.49 mm^3^ ± 2.97 mm^3^, *p* < 0.001, or a 21.8% reduction in proportional volume, *p* < 0.001; Figures 7A and 7B). In contrast, there was no change in the proportional volume of the Jz in the *Eif2s1*^*tm1RjK*^ placentas (Figure 7B). These results indicated the potential disruption of early trophoblast differentiation and placental morphogenesis in the *Eif2s1*^*tm1RjK*^ mice.

**Figure:**
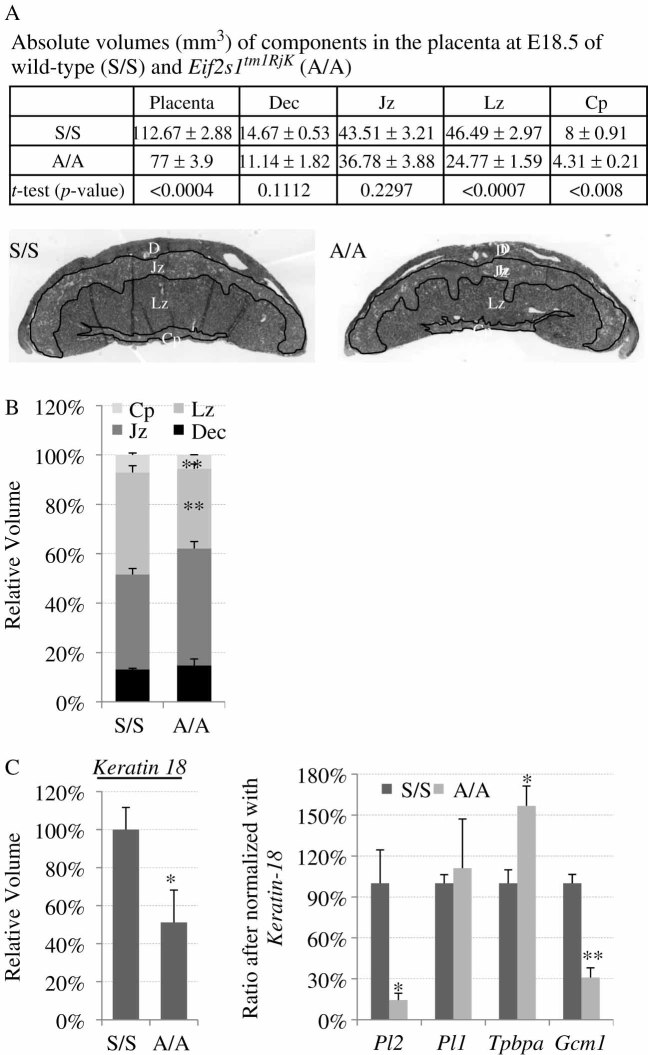
ER stress disrupts trophoblast stem cell differentiation and placental morphogenesis. (A, B) The absolute volumes and relative ratios of Lz and chorionic plate, but not of decidua and Jz, were reduced in *Eif2s1*^*tm1RjK*^ placentas. Stereological analysis was used to estimate the absolute volume of different layers. Results are presented in a table (A) and as a relative ratio in graph (B). A typical section of a placenta after H&E staining and used for stereological analysis. D = decidua; Jz = junctional zone; Lz = labyrinth zone; Cp = chorionic plate. Data are expressed as mean ± SEM from four wild-type and five *Eif2s1*^*tm1RjK*^ placentas. (C) Alteration of early trophoblast differentiation in *Eif2s1*^*tm1RjK*^ placentas. E9.5 placentas were collected and total RNA was isolated. The levels of the pan-trophoblast marker *Keratin-18*, the syncytiotrophoblast marker *Gcm1, Tpbpa, Pl1*, and *Pl2* were measured. Data are presented as mean ± SEM from five placentas in each group from two litters. All data were normalized with *Keratin-18* mRNA before expressed relative to wild-type (S/S, 100%). *p≤0.05

During murine development, placental morphogenesis occurs between E8.0 and E10.5, and formation of the labyrinth and junctional zones is complete by E14.5. Therefore, in order to identify any change in the trophoblast lineage formation, placentas were collected and analysed by quantitative RT-PCR at E9.5. At E.9.5, all *Eif2s1*^*tm1RjK*^ placentas were visually smaller than their wild-type counterparts. The early placenta is attached to a significant quantity of maternal decidua. Therefore, in order to examine the proportional contribution of different trophoblast cell types to the entire trophoblast compartment within the placenta, a pan-trophoblast lineage marker, *Keratin-18*, was used to normalize for trophoblast content. *Keratin-18* mRNA level was approximately 50% lower in *Eif2s1*^*tm1RjK*^ than in wild-type placentas (Figure 7C, left graph). After normalization, the spongiotrophoblast marker, *Tpbpa*, was significantly increased, but the giant cell marker, *Pl2*, and the syncytiotrophoblast marker, *Gcm1*, were both decreased (Figure 7C, right panel). As the spongiotrophoblast and syncytiotrophoblast are the major trophoblast cell lineages within the Jz and Lz respectively, these results are consistent with the stereological data at E18.5 demonstrating a smaller Lz, but no change in the Jz. These findings suggest that failure of early trophoblast differentiation can have long-term consequences for placental development.

## Discussion

Placental protein synthesis inhibition induced by ER stress was recently identified as a component of the pathophysiology of human IUGR (5). In this study, we sought an animal model for placental ER stress and used *Eif2s1*^*tm1RjK*^ mutant mice because they show ER stress due to their elevated basal translation rate (12). Somatic cells display a range of secretory activity. Those involved in endocrine and exocrine functions synthesize and release large quantities of proteins, and hence contain a large amount of rough ER, making them vulnerable to ER stress. Indeed, ER stress appears to be a normal physiological phenomenon in the pancreas, one of the most active endocrine and exocrine organs (9). Excessive protein synthesis has been demonstrated to aggravate ER stress in the pancreas, promoting accumulation of pro-insulin and apoptosis in β-cells, reducing circulating levels of insulin, and leading to the development of diabetes (13). Ameliorating ER stress is one of the new therapeutic interventions under investigation for the treatment of human diabetes (26).

The placenta also has a high endocrine activity. It secretes numerous hormones and growth factors that modulate maternal physiology to ensure continuance of the pregnancy and enhance the supply of nutrients, and promote its own growth through autocrine/paracrine signalling. The placentally derived insulin-like growth factors (IGFs) are particularly important for the latter and, in the mouse, Igf2 is one of the key regulators of placental size (21). Knockout of the *P0* placental-specific *Igf2* promoter results in a smaller placenta and subsequent fetal growth restriction (3). IGF2 also stimulates trophoblast proliferation and placental growth in the human (27), where again reduced placental growth precedes fetal IUGR (28). Our findings indicate that although excessive protein synthesis did not change the level of intracellular pro-Igf2, it did alter the glycosylation pattern of the secreted protein that may modulate its bioactivity (Figure 5C). Loss of function of Igf2 released from the Jz may explain the lower Pdk1–Akt–mTOR activity and reduced proliferation observed in *Eif2s1*^*tm1RjK*^ placentas through a reduction in paracrine signalling (Figure 6). Additionally, total Akt was lower in the Jz, which suffered from ER stress, but not in the labyrinth, where ER stress was not observed (Figure 6), consistent with our previous finding that ER stress attenuates Akt protein translation (15). The importance of Akt signalling in placental development is illustrated by the fact that *Akt1*-knockout mice develop an IUGR phenotype (5, 29). Interestingly, the change in placental structure and Akt signalling observed in the *Eif2s1*^*tm1RjK*^ mice is similar to the *Igf2* null placenta, with the same disproportional reduction of the Jz and Lz, and smaller placenta size (Supplementary Figure 4) (30).

All secreted and membrane-bound proteins undergo multiple post-translational modifications, such as glycosylation and disulphide bond formation, within the ER. However, under ER stress these processes are compromised, resulting in protein misfolding and potentially adversely affecting their bioactivity. Indeed, our results reveal that the glycosylation pattern of secreted proteins was changed in conditioned medium collected from *Eif2s1*^*tm1RjK*^ MEFs, and that there was an anomalous accumulation of glycoproteins in the spongiotrophoblast cells in the placental junctional zone (Figures 3 and 5A). The change in the glycoprotein profile was associated with altered bioactivity, as the *Eif2s1*^*tm1RjK*^ CM failed to maintain TS cells in a progenitor cell state. Premature differentiation of trophoblast stem/progenitor cells *in vivo* is likely to limit the expansion of the respective trophoblast compartment and to cause a relative over-abundance of post-mitotic trophoblast cell types, leading to smaller placentas, which is what we observed at E9.5. Later, at E18.5, we observed a reduction of the Lz, but not the Jz, in the mature *Eif2s1*^*tm1RjK*^ mutant placenta (Figures 7A and 7B). To exclude the possibility of a delay in implantation contributing to the smaller placenta, the number of somites, which represents the developmental stage, was counted in E9.5 embryos. No difference was found between wild-type and *Eif2s1*^*tm1RjK*^ mutants (Supplementary Table 1, Supplementary Figures 5 and 6, and Supplementary materials and methods).

To conclude, ER stress in placental endocrine cells affects the post-translational processing of secreted growth factors and other proteins, leading to altered bioactivity. Compromise of autocrine/paracrine growth factor signalling provides a mechanistic link between ER stress, trophoblast differentiation, and altered placental morphogenesis. This may be pertinent to the development of IUGR in human pregnancies, where placental ER stress is a feature of the pathophysiology. This insight may help in the development of novel therapeutic interventions targeting placental ER stress for the treatment of human IUGR.

## Author contribution statement

HWY, DSC, and GJB conceived experiments. HWY, MH, EDW, CES, and CPJ carried out experiments. RJK provided *Eif2s1*^*tm1RjK*^ mice. HWY, MH, EDW, DSC, and GJB analysed the data. HWY, DSC, and GJB were involved in writing the manuscript. All authors had final approval of the submitted and published versions.

## Supporting Information on the Internet

The following supporting information may be found in the online version of this article.

**Supplementary materials and methods.** Detailed description of materials and methods and data presented in Supplementary Table 1 and Supplementary Figures 5 and 6.

Figure S1 Images of semi-thin toluidine blue-stained sections of the Jz of wild-type (S/S) and *Eif2s1*^*tm1RjK*^ (A/A) placentas taken at 20×, 40×, and 100× magnification.

Figure S2 No change in other ER stress markers, and no increase in apoptosis in *Eif2s1*^*tm1RjK*^ placentas.

Figure S3 Low-power overview of the Jz showing the same pattern of glycoprotein staining as in Figure 3.

Figure S4 *Igf2-/*- mice display smaller placentas and fetuses, which are associated with reduced Akt signalling.

Figure S5 Litter sizes from *Eif2s1*^*tm1RjK*^ (S/A) heterozygous inter-crosses at E9.5 and E18.5.

Figure S6 *Eif2s1*^*tm1RjK*^ (A/A) mutant embryos within the normal developmental range of E9.5 were growth-restricted compared with wild-type (S/S) and *Eif2s1*^*tm1RjK*^ (S/A) embryos.

Table S1 Incidence of developmental phenotypes in conceptuses from *Eif2s1*^*tm1RjK*^ (S/A) heterozygous inter-crosses at E9.5 and E18.5.

## References

[b1] Hafner E, Metzenbauer M, Hofinger D (2003). Placental growth from the first to the second trimester of pregnancy in SGA-foetuses and pre-eclamptic pregnancies compared to normal foetuses. Placenta.

[b2] Thame M, Osmond C, Bennett F (2004). Fetal growth is directly related to maternal anthropometry and placental volume. Eur J Clin Nutr.

[b3] Constancia M, Hemberger M, Hughes J (2002). Placental-specific IGF-II is a major modulator of placental and fetal growth. Nature.

[b4] Benton SJ, Hu Y, Xie F (2012). Can placental growth factor in maternal circulation identify fetuses with placental intrauterine growth restriction?. Am J Obstet Gynecol.

[b5] Yung HW, Calabrese S, Hynx D (2008). Evidence of placental translation inhibition and endoplasmic reticulum stress in the etiology of human intrauterine growth restriction. Am J Pathol.

[b6] Schroder M, Kaufman RJ (2005). ER stress and the unfolded protein response. Mutat Res.

[b7] Harding HP, Zhang Y, Bertolotti A (2000). Perk is essential for translational regulation and cell survival during the unfolded protein response. Mol Cell.

[b8] Nika J, Rippel S, Hannig EM (2001). Biochemical analysis of the eIF2beta gamma complex reveals a structural function for eIF2alpha in catalyzed nucleotide exchange. J Biol Chem.

[b9] Iwawaki T, Akai R, Kohno K (2004). A transgenic mouse model for monitoring endoplasmic reticulum stress. Nature Med.

[b10] Iwawaki T, Akai R, Yamanaka S (2009). Function of IRE1 alpha in the placenta is essential for placental development and embryonic viability. Proc Natl Acad Sci U S A.

[b11] Harding HP, Zeng H, Zhang Y (2001). Diabetes mellitus and exocrine pancreatic dysfunction in perk − /− mice reveals a role for translational control in secretory cell survival. Mol Cell.

[b12] Scheuner D, Song B, McEwen E (2001). Translational control is required for the unfolded protein response and *in vivo* glucose homeostasis. Mol Cell.

[b13] Scheuner D, Mierde D, Song B (2005). Control of mRNA translation preserves endoplasmic reticulum function in beta cells and maintains glucose homeostasis. Nature Med.

[b14] Oslowski CM, Urano F (2011). The binary switch that controls the life and death decisions of ER stressed beta cells. Curr Opin Cell Biol.

[b15] Yung HW, Korolchuk S, Tolkovsky AM (2007). Endoplasmic reticulum stress exacerbates ischemia–reperfusion-induced apoptosis through attenuation of Akt protein synthesis in human choriocarcinoma cells. FASEB J.

[b16] Jones CJ, Aplin JD, Burton GJ (2010). First trimester histiotrophe shows altered sialylation compared with secretory phase glycoconjugates in human endometrium. Placenta.

[b17] Coan PM, Ferguson-Smith AC, Burton GJ (2004). Developmental dynamics of the definitive mouse placenta assessed by stereology. Biol Reprod.

[b18] Singh I, Doms RW, Wagner KR (1990). Intracellular transport of soluble and membrane-bound glycoproteins: folding, assembly and secretion of anchor-free influenza hemagglutinin. EMBO J.

[b19] Tanaka S, Kunath T, Hadjantonakis AK (1998). Promotion of trophoblast stem cell proliferation by FGF4. Science.

[b20] Hughes M, Dobric N, Scott IC (2004). The Hand1, Stra13 and Gcm1 transcription factors override FGF signaling to promote terminal differentiation of trophoblast stem cells. Dev Biol.

[b21] Roberts CT, Owens JA, Sferruzzi-Perri AN (2008). Distinct actions of insulin-like growth factors (IGFs) on placental development and fetal growth: lessons from mice and guinea pigs. Placenta.

[b22] Valenzano KJ, Heath-Monnig E, Tollefsen SE (1997). Biophysical and biological properties of naturally occurring high molecular weight insulin-like growth factor II variants. J Biol Chem.

[b23] DeChiara TM, Efstratiadis A, Robertson EJ (1990). A growth-deficiency phenotype in heterozygous mice carrying an insulin-like growth factor II gene disrupted by targeting. Nature.

[b24] Tamai Y, Nakajima R, Ishikawa T (1999). Colonic hamartoma development by anomalous duplication in Cdx2 knockout mice. Cancer Res.

[b25] Guzman-Ayala M, Ben-Haim N, Beck S (2004). Nodal protein processing and fibroblast growth factor 4 synergize to maintain a trophoblast stem cell microenvironment. Proc Natl Acad Sci U S A.

[b26] Engin F, Hotamisligil GS (2010). Restoring endoplasmic reticulum function by chemical chaperones: an emerging therapeutic approach for metabolic diseases. Diabetes Obes Metab.

[b27] Harris LK, Crocker IP, Baker PN (2010). IGF2 actions on trophoblast in human placenta are regulated by the insulin-like growth factor 2 receptor, which can function as both a signaling and clearance receptor. Biol Reprod.

[b28] Proctor LK, Toal M, Keating S (2009). Placental size and the prediction of severe early-onset intrauterine growth restriction in women with low pregnancy-associated plasma protein-A. Ultrasound Obstet Gynecol.

[b29] Yang ZZ, Tschopp O, Hemmings-Mieszczak M (2003). Protein kinase B alpha/Akt1 regulates placental development and fetal growth. J Biol Chem.

[b30] Coan PM, Fowden AL, Constancia M (2008). Disproportional effects of Igf2 knockout on placental morphology and diffusional exchange characteristics in the mouse. J Physiol.

[b31] Watson ED, Cross JC (2005). Development of structures and transport functions in the mouse placenta. Physiology (Bethesda).

