# Supplementary figures and images for: Endoplasmic reticulum stress disrupts placental morphogenesis: implications for human intrauterine growth restriction
Source: J Pathol. 2012 Sep 28;228(4):554–64. doi: 10.1002/path.4068 (PMC3532660; doi:10.1002/path.4068)

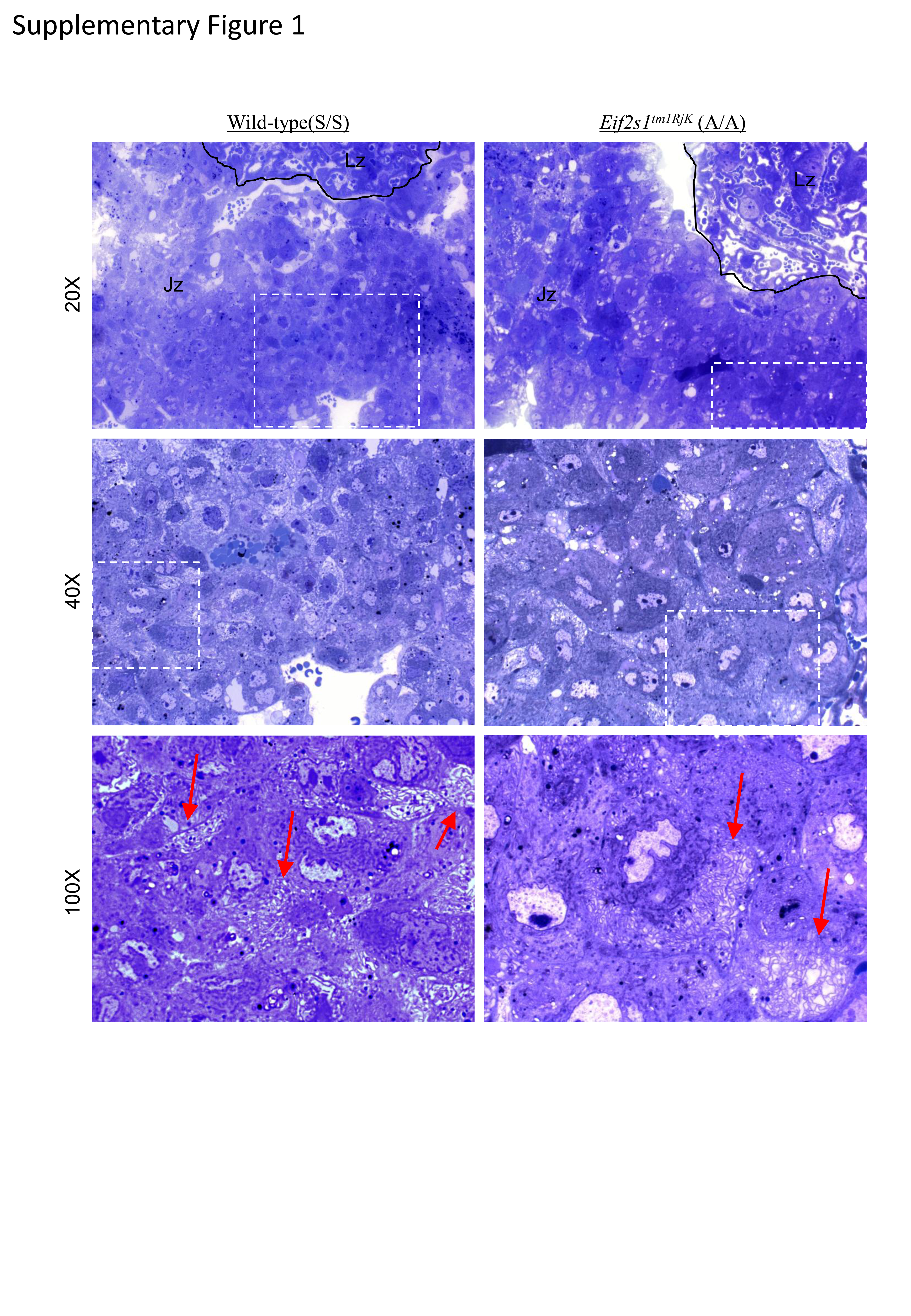

Supplement: Figure S1 — Images of semi-thin toluidine blue-stained sections of the Jz of wild-type (S/S) and Eif2s1tm1RjK (A/A) placentas taken at 20×, 40×, and 100× magnification. [file path0228-0554-SD1.tif]

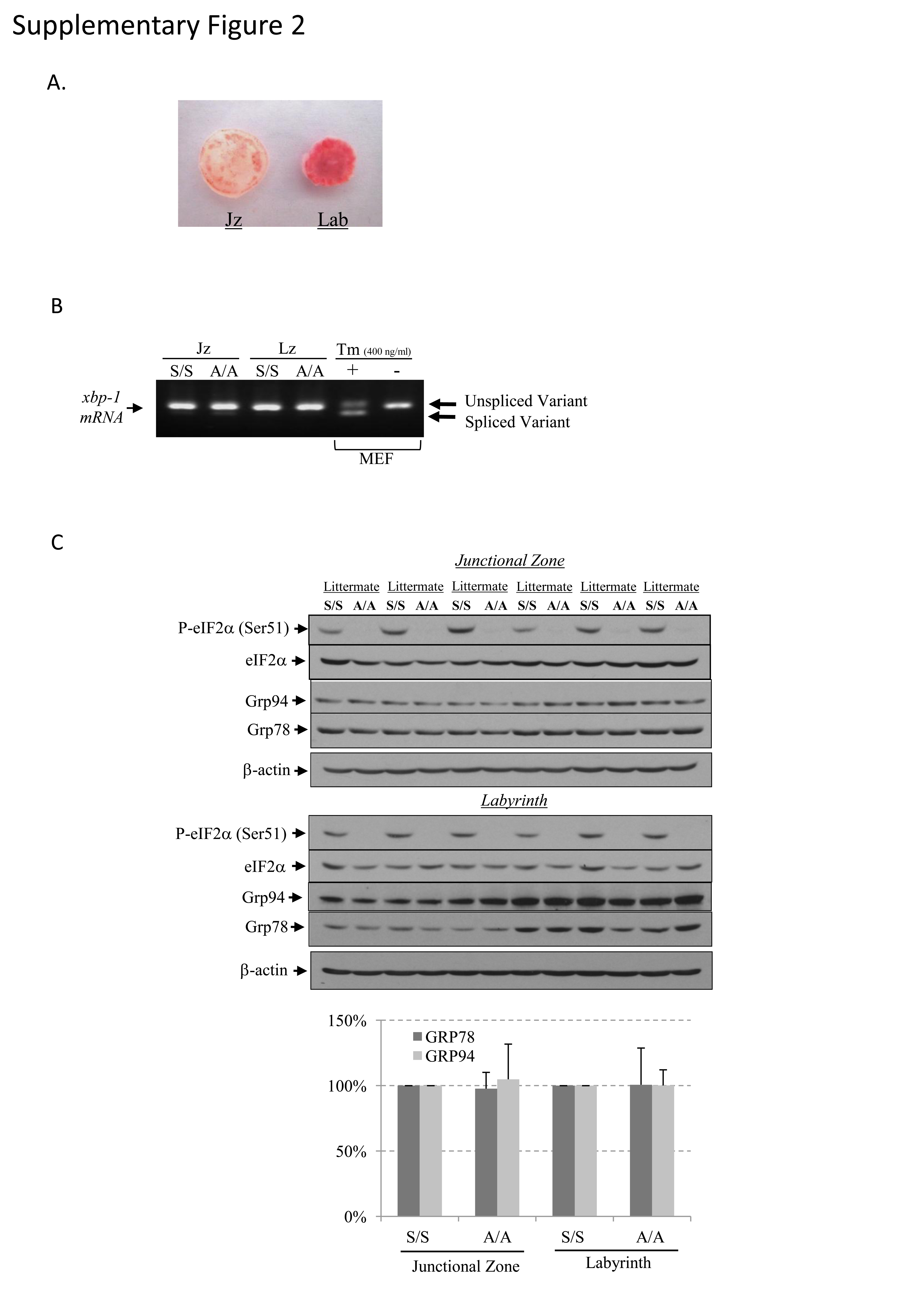

Supplement: Figure S2 — No change in other ER stress markers, and no increase in apoptosis in Eif2s1tm1RjK placentas. [file path0228-0554-SD2.tif]

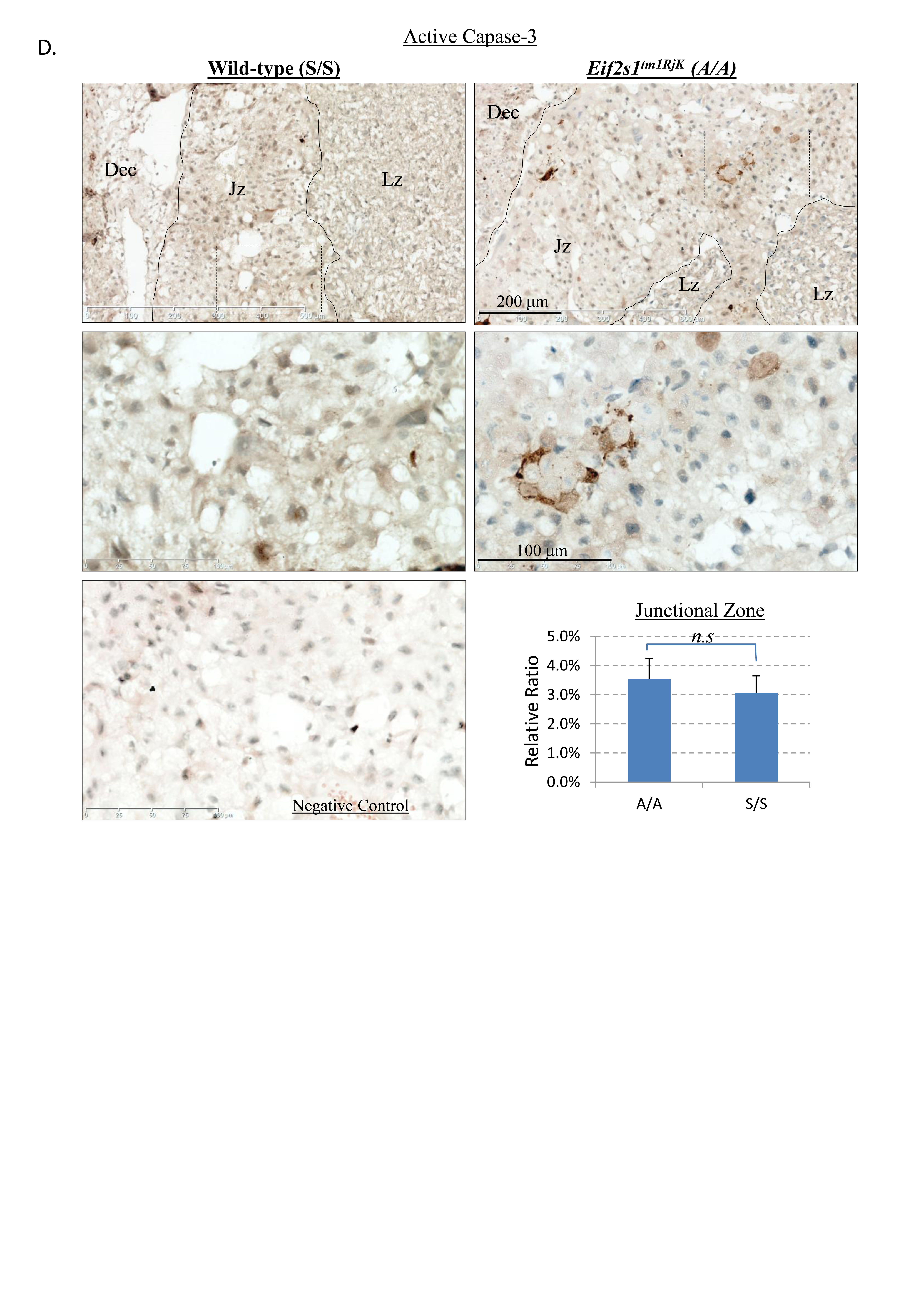

Supplement: Figure S2 — No change in other ER stress markers, and no increase in apoptosis in Eif2s1tm1RjK placentas. [file path0228-0554-SD3.tif]

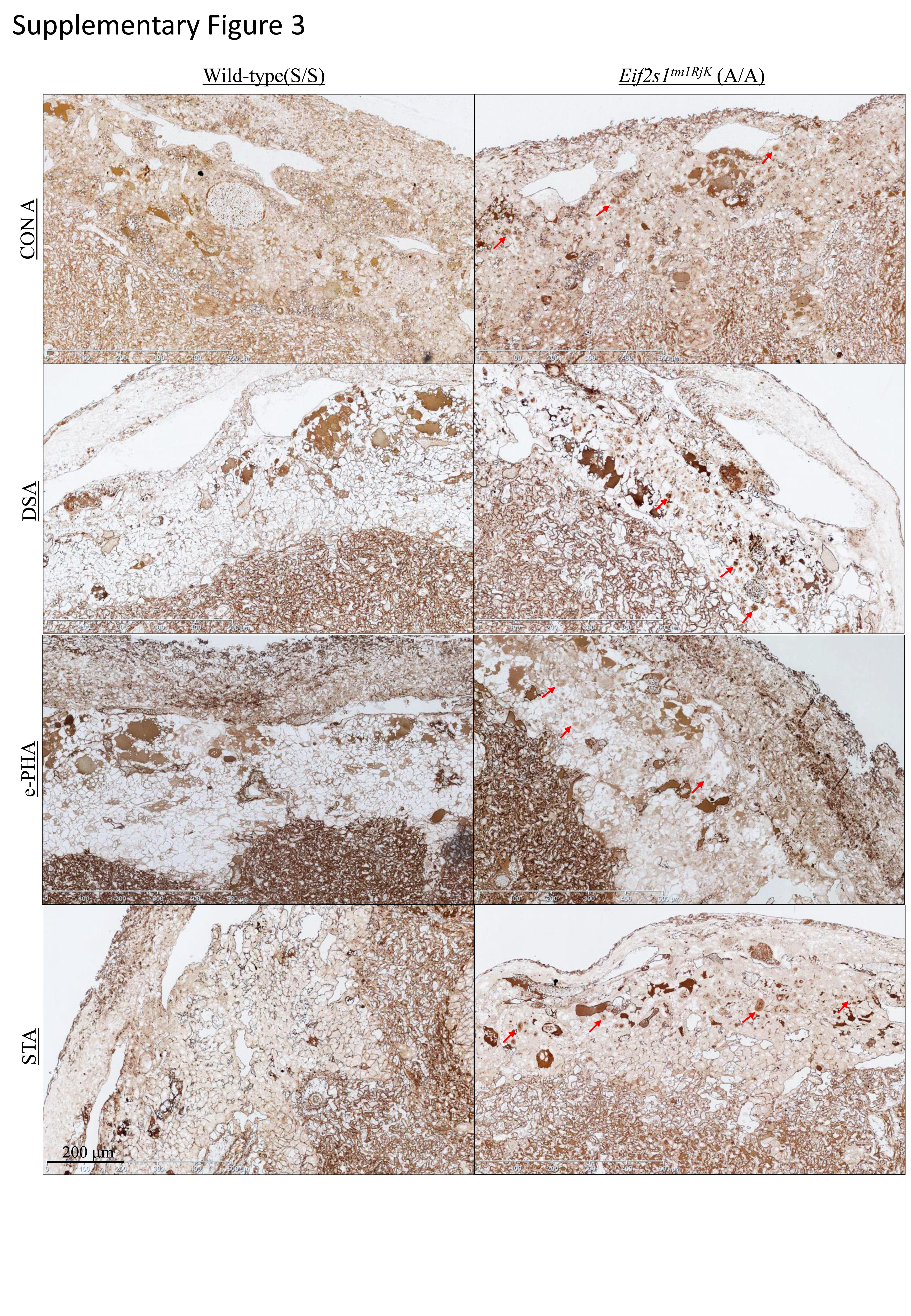

Supplement: Figure S3 — Low-power overview of the Jz showing the same pattern of glycoprotein staining as in Figure 3. [file path0228-0554-SD4.tif]

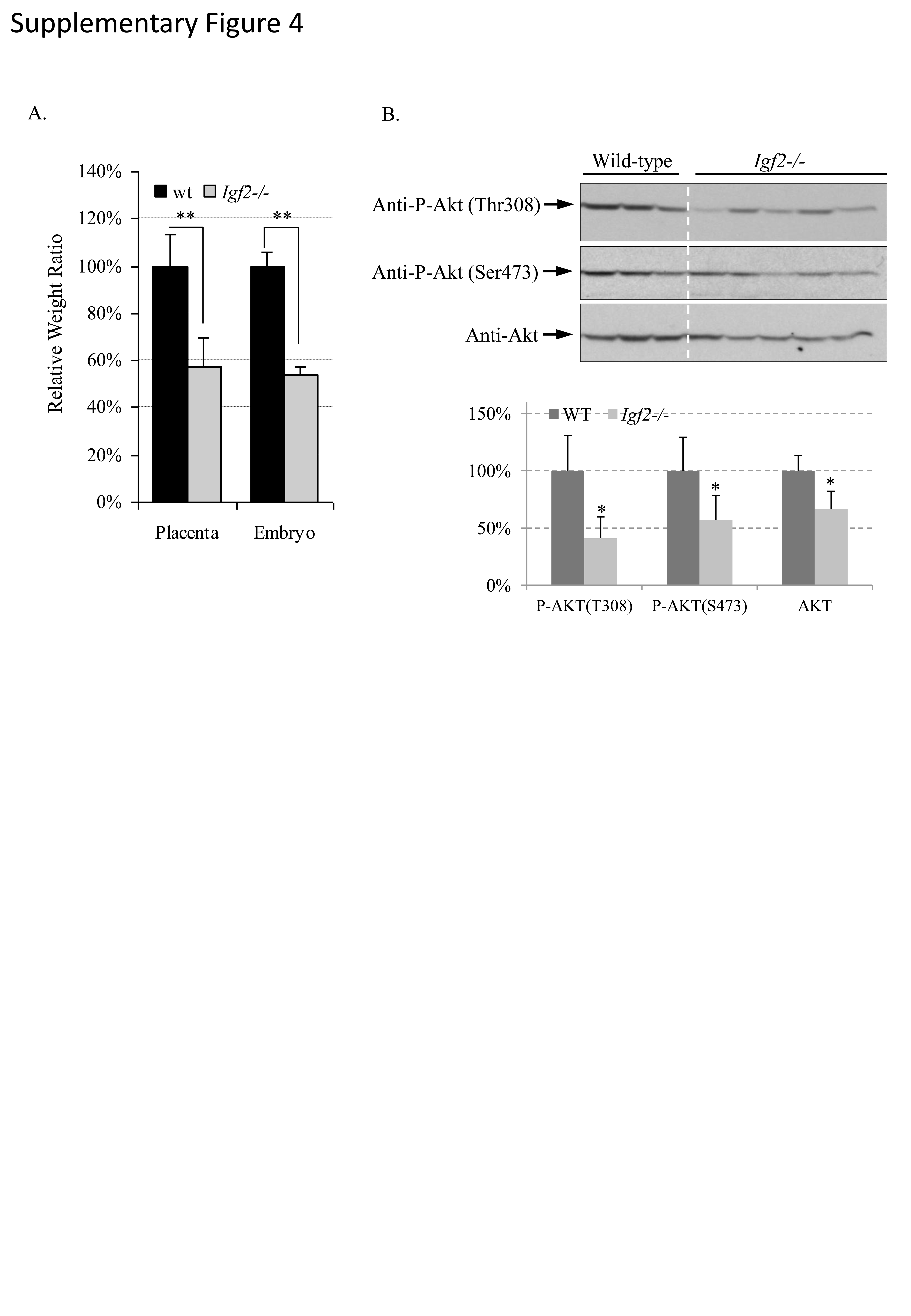

Supplement: Figure S3 — Low-power overview of the Jz showing the same pattern of glycoprotein staining as in Figure 3. [file path0228-0554-SD5.tif]

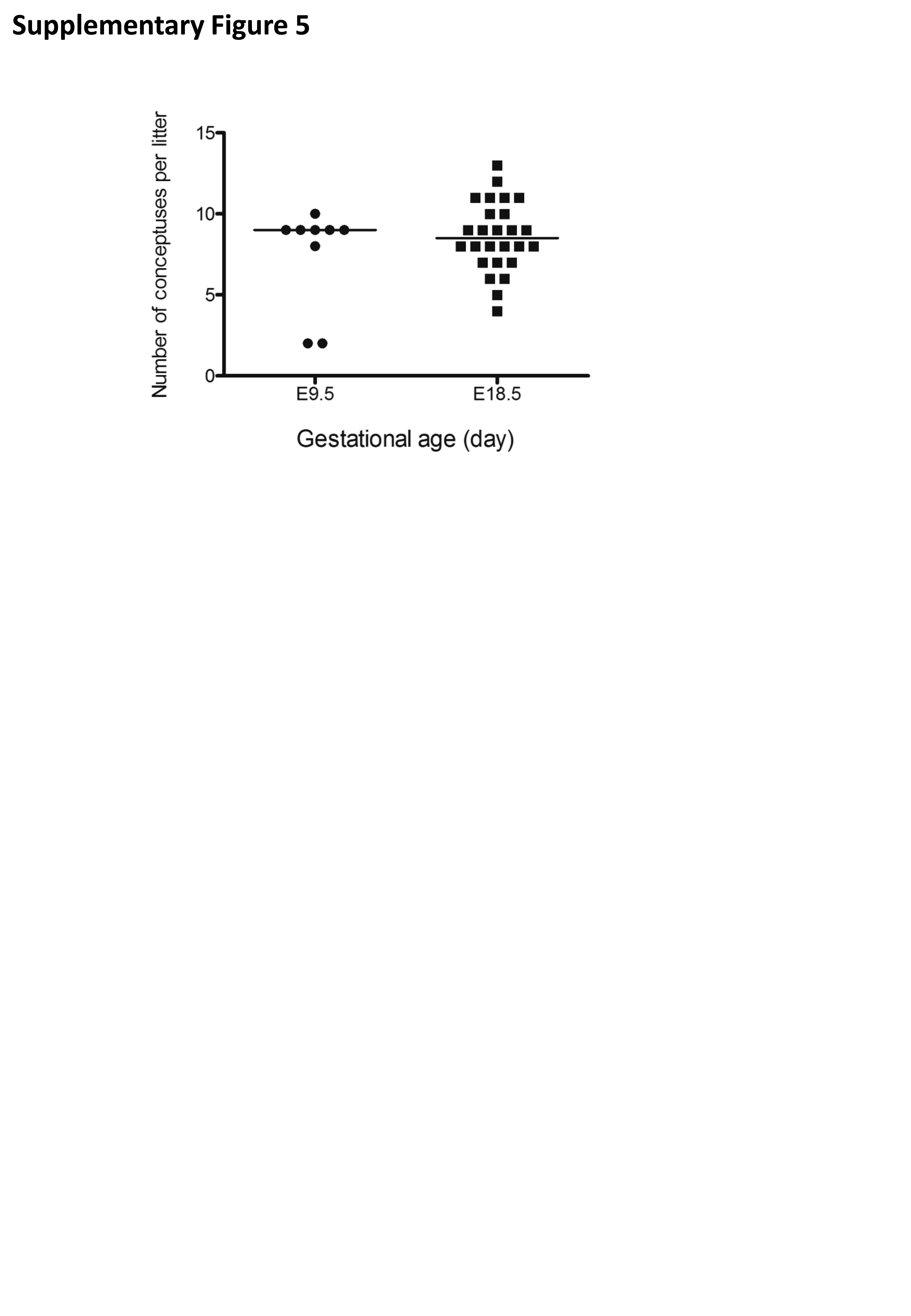

Supplement: Figure S4 — Igf2-/- mice display smaller placentas and fetuses, which are associated with reduced Akt signalling. [file path0228-0554-SD6.tif]

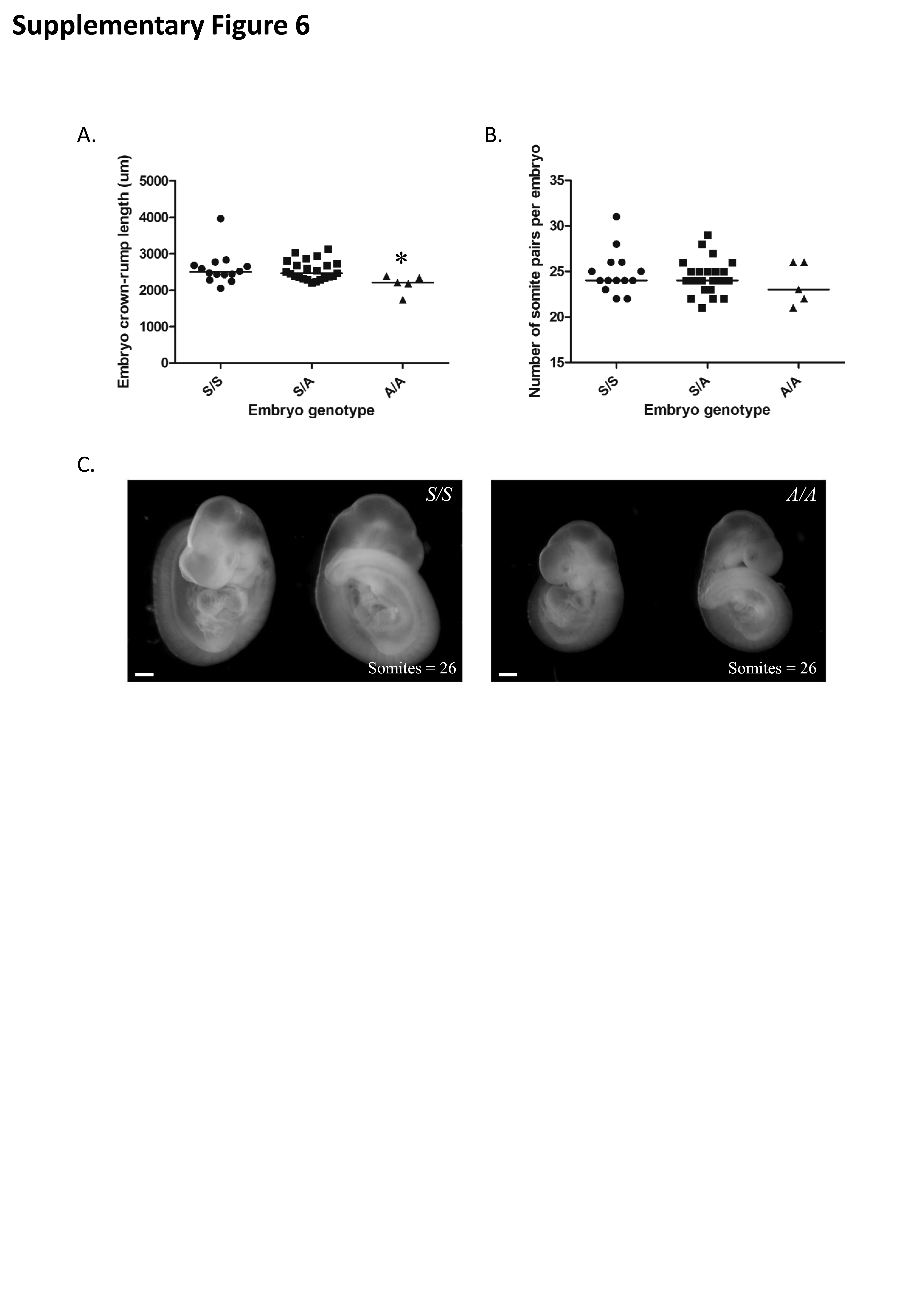

Supplement: Figure S6 — Eif2s1tm1RjK (A/A) mutant embryos within the normal developmental range of E9.5 were growth-restricted compared with wild-type (S/S) and Eif2s1tm1RjK (S/A) embryos. [file path0228-0554-SD8.tif]

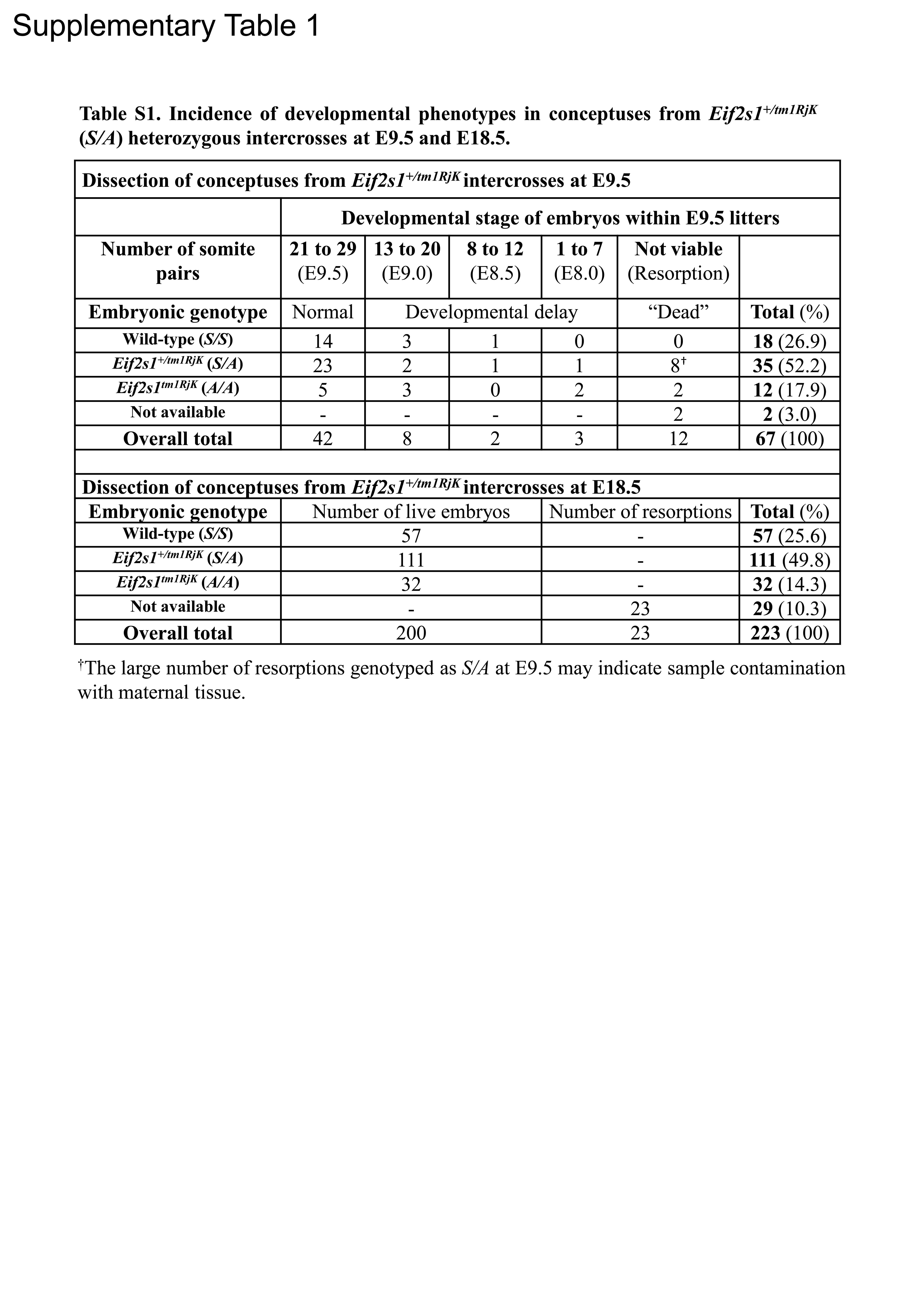

Supplement: Table S1 — Incidence of developmental phenotypes in conceptuses from Eif2s1tm1RjK (S/A) heterozygous inter-crosses at E9.5 and E18.5. [file path0228-0554-SD9.tif]
